# Grip on complexity in chemical reaction networks

**DOI:** 10.3762/bjoc.13.147

**Published:** 2017-07-28

**Authors:** Albert S Y Wong, Wilhelm T S Huck

**Affiliations:** 1 Institute for Molecular Materials, Radboud University Nijmegen, Heyendaalseweg 135, 6525 AJ Nijmegen, The Netherlands

**Keywords:** chemical reaction network, complexity, dissipative systems, network motifs, out-of-equilibrium, tunability

## Abstract

A new discipline of “systems chemistry” is emerging, which aims to capture the complexity observed in natural systems within a synthetic chemical framework. Living systems rely on complex networks of chemical reactions to control the concentration of molecules in space and time. Despite the enormous complexity in biological networks, it is possible to identify network motifs that lead to functional outputs such as bistability or oscillations. To truly understand how living systems function, we need a complete understanding of how chemical reaction networks (CRNs) create function. We propose the development of a bottom-up approach to design and construct CRNs where we can follow the influence of single chemical entities on the properties of the network as a whole. Ultimately, this approach should allow us to not only understand such complex networks but also to guide and control their behavior.

## Review

### Introduction

Natural phenomena, such as the earth’s climate, ecosystems, animal group behavior, our brain, and living cells, are all systems that display dynamic behavior marked by an apparent complexity [1–5]. Some of the remarkable properties of complex systems lie in their robustness (i.e., error tolerance), resilience (i.e., restoration ability) and adaptive capacity (i.e., to compete or to cooperate for resources) in response to changes in environmental conditions [6–11]. Such system-level functions represent the prerequisite in natural phenomena to prevent abrupt climate shifts or the sudden diminishing of populations in ecosystems and are, arguably, the key properties supporting complex systems to transition from non-living to living [12–14]. Understanding the principles enabling transitions between dynamically distinct but stable states will unravel the predictability and perhaps the possibility to influence the dynamics of change, but science has yet to find an answer to this complexity [15].

One of the ultimate aims for systems like a living cell, is to understand how the interplay between molecular level events and network topology determines the behavior that emerges from complex networks of chemical reactions [16]. Vast metabolic and genetic networks of chemical reactions allow living cells to sense their environment, react to stimuli, and use nutrients for cell growth and division. In the past decades, complexity science has made tremendous progress in developing mathematical tools that capture the key properties underlying such networks [17–19]. Our in-depth knowledge of actual systems, however, is often insufficient to precisely predict when, and by how much, systems respond to changes in the environment. This is especially true when those changes induce systems beyond a critical value, where the resulting abrupt shifts or phase transitions become unpredictable [20–22]. The analysis of the structure and the dynamics of a complex web of intricate interactions is a risk in removing the link between molecular structure and function and network behavior. Hence, we need new approaches that allow guidance and ultimately control of unanticipated behavior of complex molecular systems.

Networks are daunting in complexity but do exhibit structural patterns [23]. The reduction of a network into wiring diagrams enables accurate modelling and has revealed fundamental features that would otherwise be too difficult to comprehend [24]. It is generally accepted that complex molecular networks, like electrical circuits, are constructed from simpler modules (network motifs) and control the regulatory functions as well as the system level behavior of larger networks [25]. In fact, simple motifs with a few positive and negative feedback loops create functionality, such as bistable switching, adaptation and oscillations [26]. Such building blocks can be reconstructed, and this has sparked enormous activity in the fields of synthetic and systems biology as well as metabolic engineering [27].

We must now learn how to apply retrosynthesis to network motifs, and we believe chemistry offers a unique opportunity to the design of chemical reaction networks (CRNs) [28–30]. A major challenge for systems chemistry is to translate the design principles of biological systems into a practical “programming language” and learn how to create functionality using chemical reactions. Early work has resulted in numerous exciting examples, ranging from functional out-of-equilibrium systems that can perform logic operations, to dissipative self-assembling structures, creating new forms of smart materials [31–35]. Yet, we are severely limited by too few examples of systems which are both extensive enough to exhibit dynamics, and at the same time, simple enough to be tunable [36].

In this perspective, we will attempt to lay out a general strategy for the design and implementation of CRNs that operate under out-of-equilibrium conditions and show complex behavior. We believe that new approaches are needed to build molecular networks, firmly rooted in (synthetic) chemistry but incorporating mathematical modelling and borrowing principles from chemical engineering [37]. Isolating the influence of molecular structure on network function and dynamics will reveal the rules governing CRNs, as well as the complexity in systems like the cell.

### Minimal chemical reaction networks

#### Network motifs assembled from feedback loops

Much of our inspiration for constructing CRNs comes from the living cell [38–41]. The biochemical network that governs the dynamic properties of physiological responses such as growth, division and death, can be depicted as a wiring diagram [42–43]. Despite the large number of possible connections, certain patterns of interconnections, so-called “network motifs”, are relatively common [23]. Hence, underneath the complexity, local regulation based on minimal systems comprises a fairly simple set of basic events (i.e., activation and inhibition).

**Figure 1:**
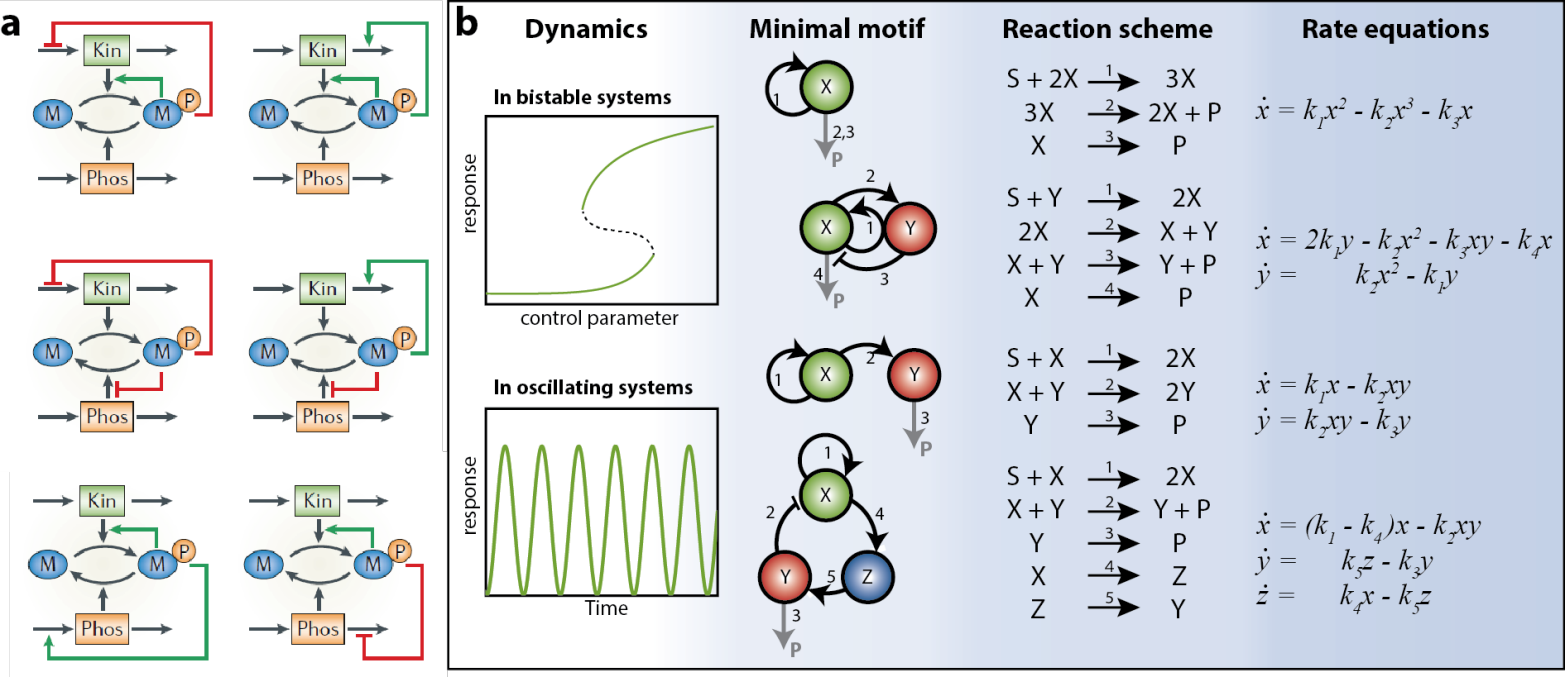
Network motifs. (a) Examples of network motifs composed from different feedback loops. Each design can turn a universal signaling cycle into a bistable switch and relaxation oscillator. Adapted with permission from [24], copyright 2006 Nature Publishing Group. (b) Minimal network motifs that describe the dynamics of minimal bistable or oscillatory systems using mass-action kinetics [49].

Minimal network motifs have the advantage of being simple enough (i.e., analytically solvable) and are therefore well-suited for approaches viewed in the framework of rates of chemical reactions. Figure 1a shows more detail on how a simple phosphorylation and dephosphorylation system can influence the rates of its own formation creating either a positive and/or a negative feedback loop [24,26] Through feedback loops, even simple systems composed from minimal motifs can display dynamic behavior. A minimum of one positive feedback structurally promotes bistability in networks [44] but additional interactions linking of the activation and inhibition provide the necessary nonlinearity to stabilize the on/off state [45–48]. Figure 1b depicts several examples of motifs that are considered responsible for the regulatory functions that generate discontinuous bistable dynamics or oscillations [49].

#### Network motifs are dynamic building blocks

Network motifs, like bistable switches and oscillators, form the basic building blocks of dynamic behavior. A common approach to understand the underlying biological phenomena uses a mathematical model that consists of coupled nonlinear ordinary differential equations (ODEs) [50–53]. Feedback loops, in fact, are simply interactions based upon elementary mono- and bimolecular chemical reactions that are subject to the same chemical laws as classical reactions [54]. As such, the motifs summarized in Figure 1b can be translated into stoichiometric reaction schemes. Under the assumption of spatially homogeneous conditions, the dynamics can be fully described by the rate equations in the subsequent column [49,55].

The practical realization of dynamic properties in such reaction schemes is daunting in part because it also requires the systems to operate far from equilibrium [56–57]. A venture beyond the confines of equilibrium, however, does not require deeper understanding of the thermodynamic laws in nonequilibrium systems. Open systems allow environmental conditions to influence the accessibility or stability of the final state, marking the key difference between systems *in* and *out* of equilibrium [58]. Although their behavior is only predictable by a full understanding of the exact ensemble of rate equations, the steady state solutions satisfy the same algebraic equation that controls equilibrium state solutions: 0 = d*x*/d*t* = d*y*/d*t* (= d*z*/d*t*) [59]. To keep the chemical system from reaching equilibrium (i.e., in a thermodynamically open system), the implementation of CRNs often suffices with the assumption of an excess of a source (S) combined with a product (P) that acts as a sink. In such dissipative conditions, reactions do not necessarily settle for the state with the highest entropy but instead are drawn towards a steady state.

### From network motifs to dissipative systems

#### Classical example of a chemical dissipative system

**Figure 2:**
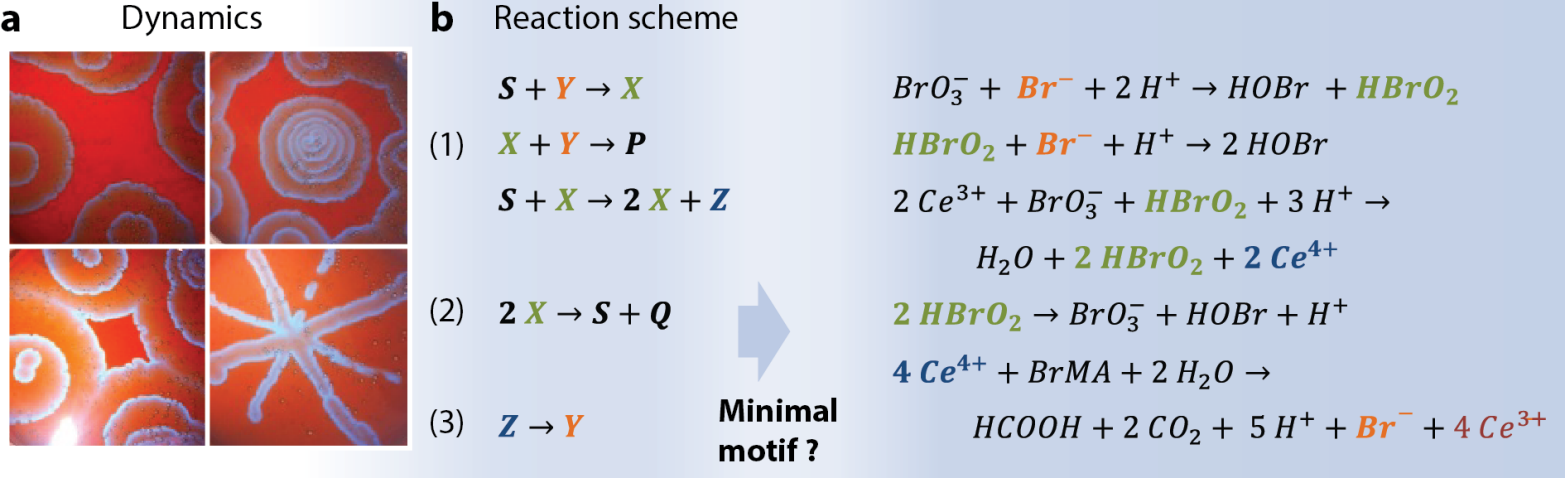
Belousov–Zhabotinsky (BZ) reaction. (a) Classical example of pattern formation in the BZ reaction when perturbed with a silver thread. Adapted with permission from [60], copyright 1970 Nature Publishing Group. (b) The multitude of reactions in the BZ reaction can be reduced to the Oregonator, a three-variable scheme (with key species X = HBrO_2_, Y = Br^−^, and Z = Ce^4+^) [68–69].

Network motifs can guide the design of CRNs, but first, we need to develop an intuition for the components that make up a network motif. The Belousov–Zhabotinsky (BZ) oscillating reaction is arguably the best-known chemical network (Figure 2a) [60]. As a prototypical out-of-equilibrium system, the BZ reaction provided the fundamental and experimental basis for nonlinear chemistry [61–64]. Studies as diverse as synchronization in coupled systems, oscillatory Turing patterns, and spatio-temporal chaos show that the rich dynamics depend solely on how energy dissipates from the system, initiated by local instabilities [65–67].

We must learn how to apply retrosynthesis to chemical reaction networks such as the BZ reaction. The reaction scheme in Figure 2b shows that the BZ network comprises five reactions that can be translated into three inorganic processes in acidic conditions: (1) autocatalytic production of HOBr_2_ (X) in the presence of Br^−^ (Y), (2) oxidation of the cerium catalyst, Ce^3+^ → Ce^4+^ (Z), and (3) the regeneration of Br^−^ and Ce^3+^ fueled by the oxidation of malonic acid (MA) [68–69]. Translation from the reaction scheme or equations (back) to the network motif, however, is far from intuitive. Hence, despite its beauty and obvious potential for making exciting discoveries, the BZ reaction (and similar classical chemical systems [70–73]) lack bottom-up design opportunities. Furthermore, the incorporation of a wider range of (organic) chemical reactions is challenging due to the aggressive nature of the medium and reactants.

#### Chemical dissipative systems based on tunable organic structures

**Figure 3:**
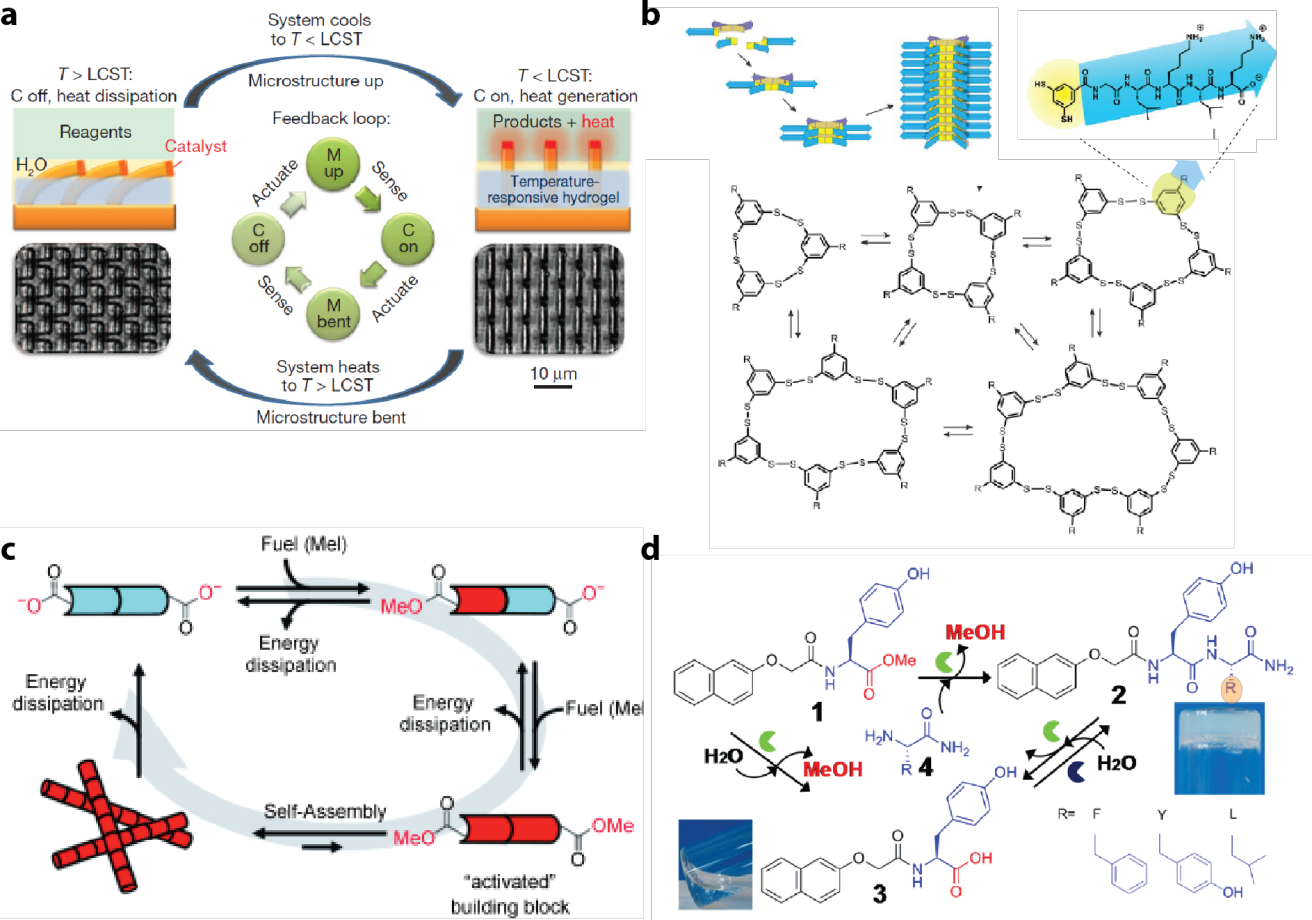
Examples of synthetic dissipative systems. (a) Feedback cycle of a bilayer network composed of the mechanical action of a temperature-responsive gel coupled with various exothermic reactions. Reprinted with permission from [34], copyright 2012 Nature Publishing Group. (b) Small dynamic combinatorial library made from dithiol building blocks. Adapted with permission from [75], copyright 2013 American Chemical Society. (c) Self-assembly fibrous structures fueled by molecular gelators. Reprinted with permission from [76], copyright 2010 Wiley-VCH Verlag GmbH & Co. (d) Biocatalytic self-assembly in the presence of chymotrypsin (green) forming hydrogelators that can be modified by the choice of amino acids depicted in the bottom right side. Reprinted with permission from [77], copyright 2013 American Chemical Society.

The more recent work is focused on building chemical dissipative networks from organic structures. This has resulted in numerous exciting examples, ranging from functional out-of-equilibrium systems that can perform logic operations to dissipative self-assembling structures, creating new forms of smart materials (Figure 3a–d) [31–35,74–77]. The underlying principle of compartmentalization, dynamic combinatorial chemistry, and hydrogelation also appears in different types of networks [78–84]. Chemical networks can be readily made from tunable organic structures, holding considerable potential in the chemical sciences for the development of a new approach to the construction of out-of-equilibrium molecular networks.

A remaining key challenge encountered in the experimental realization of robust steady-state output in such systems is to balance the reaction rates between various feedback loops in the network. Despite the advances made, the behavior in reactions while approaching equilibrium (Figure 3c,d) is transient and not a reflection of a dynamic steady state. Hence, the bottom-up construction of chemical reaction networks requires more than convenient chemical components. A general methodology is needed that integrates the thorough appreciation of reaction rates in the design of chemical networks.

#### Learning from the design principles applied in synthetic biology

**Figure 4:**
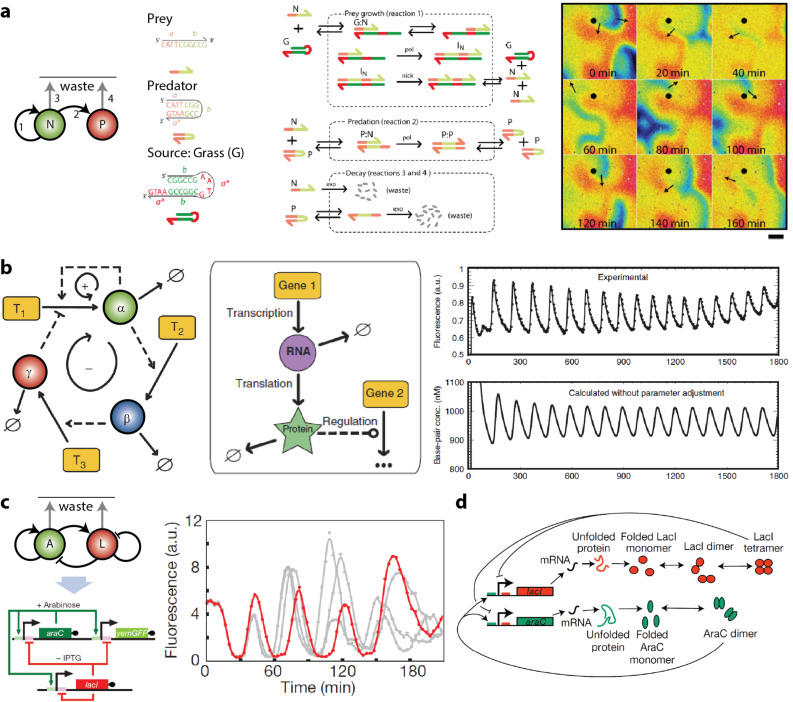
Design principles applied in synthetic biology. (a) Network topology, mechanism, and the clockwise-rotating spiral of the prey in the molecular predator–prey network. Adapted with permission from [88], copyright 2013 American Chemical Society. (b) The gene regulation pathway and of an oscillator based on a positive and delayed negative feedback motif, with experimentally observed oscillations shown to be in good agreement with the simulations. Adapted with permission from [90], copyright 2011 the authors. (c) Oscillations in the dual-feedback motif. (d) Illustration of the explicit intermediate processes required for accurate simulations in the mathematical modelling of genetic reaction networks. Adapted with permission from [91], copyright 2008 Nature Publishing Group.

Genetic and small DNA-oligonucleotide networks provide an ideal test bed to address the basic principles of designing (bio)chemical complex systems [85–87]. Figure 4a shows the successful translation of an earlier discussed network motif into a molecular predator (P)–prey (N) network [88–89]. The information concerning the predator and prey growth and degradation is stored in single-stranded DNA (ssDNA). Importantly, the reaction scheme and rate equations could be approximated based on the predictability in the thermodynamics of DNA binding. In presence of an excess of the source ssDNA (denoted by G for “Grass”), traveling waves of a predator–prey molecular network (similar to the spatio-temporal patterns in the BZ reaction) were obtained. In stark contrast to the BZ reaction, however, the use of DNA or DNA-enzyme-based in vitro systems are amenable to rational design.

Other approaches in synthetic biology use gene regulatory networks. Gene regulations provide both conceptual simplicity and modularity to design networks exhibiting oscillatory behavior. Within this framework, Figure 4b shows an in vitro implementation of an oscillator comprising a positive and a delayed negative feedback loop [90]. The canonical gene regulation pathway uses the information encoded in DNA templates T_1_−T_3_. Similarly, a genetic oscillator can be engineered in *Escherichia coli* [85–87]. Figure 4c shows the network composed of AraC, LacI and yemGFP genes [91]. The additional yemGFP gene serves as a read-out component and is not depicted in the regulatory network motif.

Together, the examples in Figure 4 demonstrate that complex dynamics could be achieved by transcription and translation processes. Dissipation arises from an approximated constant supply of nucleotides, amino acids, and enzymes among other cellular machinery (see Figure 4d) [92]. Arguably, the ability to rationally assemble test tube CRNs lags behind that for in vivo systems due to difficulties faced in mimicking such cellular composition [93]. Consequently, in molecular “circuits” based on DNA as building blocks, certain reaction rates are often not known, cannot be known, or cannot be tuned easily.

### Blueprint for the construction of chemical complex systems

**Figure 5:**
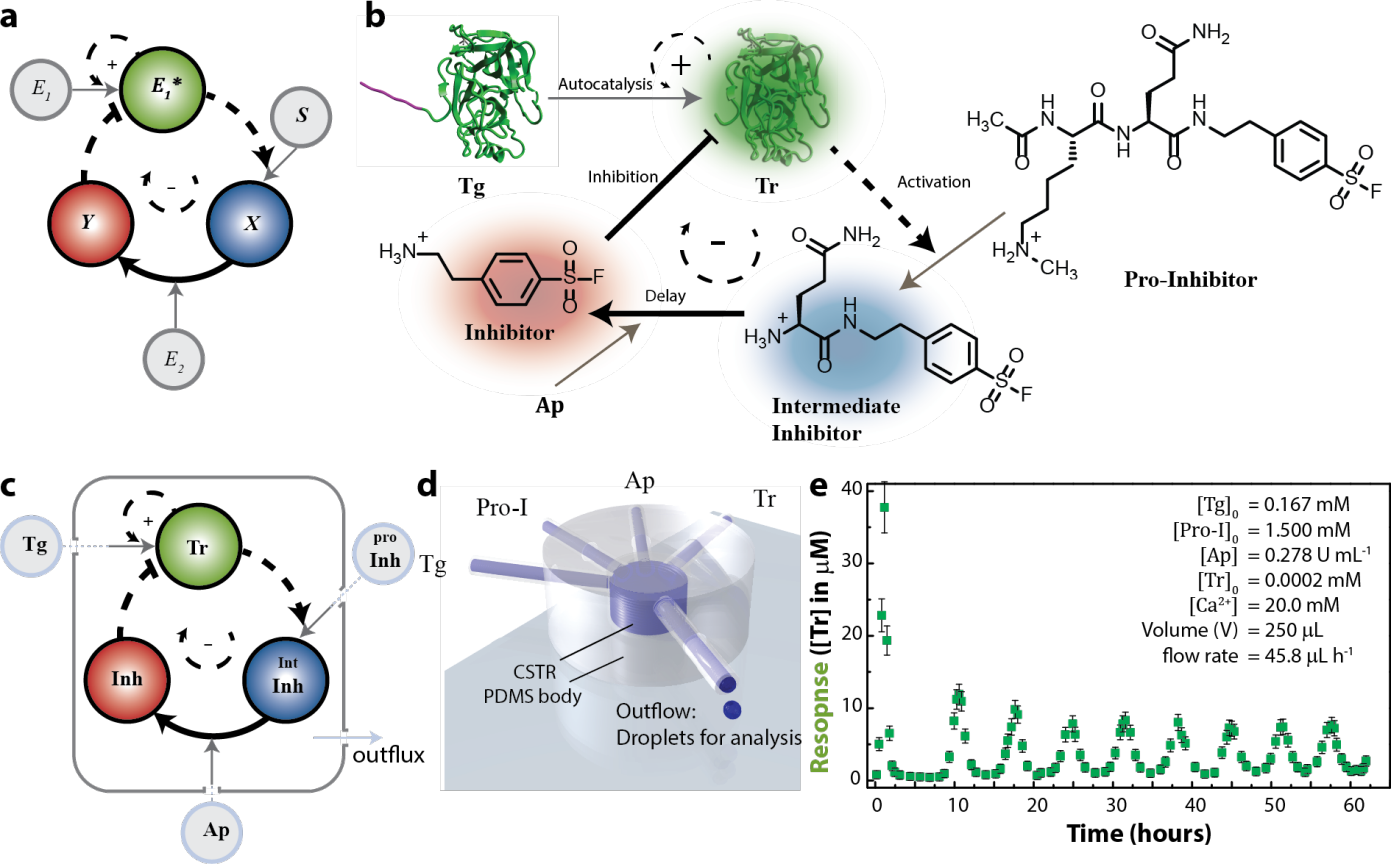
A retrosynthetic design strategy to implement an oscillating enzymatic reaction network [94]. (a) Schematic network layout of the enzymatic oscillator based on a delayed negative feedback motif. (b) Detailed reaction diagram of the CRN. (c) Illustration of the central network motif based on autocatalytic production and delayed inhibition of an enzyme that was kept out-of-equilibrium. (d) Schematic representation of the flow reactor that is used to implement flow. (e) The output of the reactor was measured using an enzymatic activity in an offline analysis. The response shows oscillations in Tr activity (with experimental conditions in inset). Adapted with permission from [94], copyright 2015 Nature Publishing Group.

A chemical approach, in contrast to synthetic biology, might involve the construction of a network of individual reactions that are well-characterized where the key kinetic parameters can all be experimentally verified. We recently showed that a chemical reaction network can be designed using enzymatic reactions combined with the tuning of the reaction rates in (small) molecules [94]. The initial point of the design was to select a network motif for which the steady state output is known. Our network combines a positive and a delayed negative feedback loop (Figure 5a) that is built around a key enzyme E_1_*. In the reaction network, trypsin (Tr) catalyzes its own formation from the precursor trypsinogen (Tg). Opposed to this positive feedback, Tr is inhibited by the negative feedback that is composed of three sequential steps (Figure 5b). In the activation step, Tr converts a pro-inhibitor into an intermediate inhibitor (Int-Inh), which consists of a glutamine (Gln) residue attached to a potent inhibitor for Tr. Another enzyme, aminopeptidase N (Ap), controls the release of the inhibitor moiety by cleaving off Gln in the delay step. In the final step, Tr recognition of the active inhibitor (Inh) closes the negative feedback loop.

The network displays complex behavior in an open system. In contrast to earlier examples, we used a continuous flow of the reactants (Tg, Ap, and Pro-I) to create out-of-equilibrium conditions (Figure 5c) [95]. A poly(dimethylsiloxane) (PDMS)-based microfluidic continuous stirred tank reactor (CSTR) was conveniently prepared in which the flow (i.e., the reciprocal of the residence time defined by the ratio of the outflux and the reactor volume) maintained out-of-equilibrium conditions for the system (Figure 5d). The response of the system is determined by the concentration of the time course of Tr. Figure 5e demonstrates that the CRN is capable of producing sustained oscillations.

**Figure 6:**
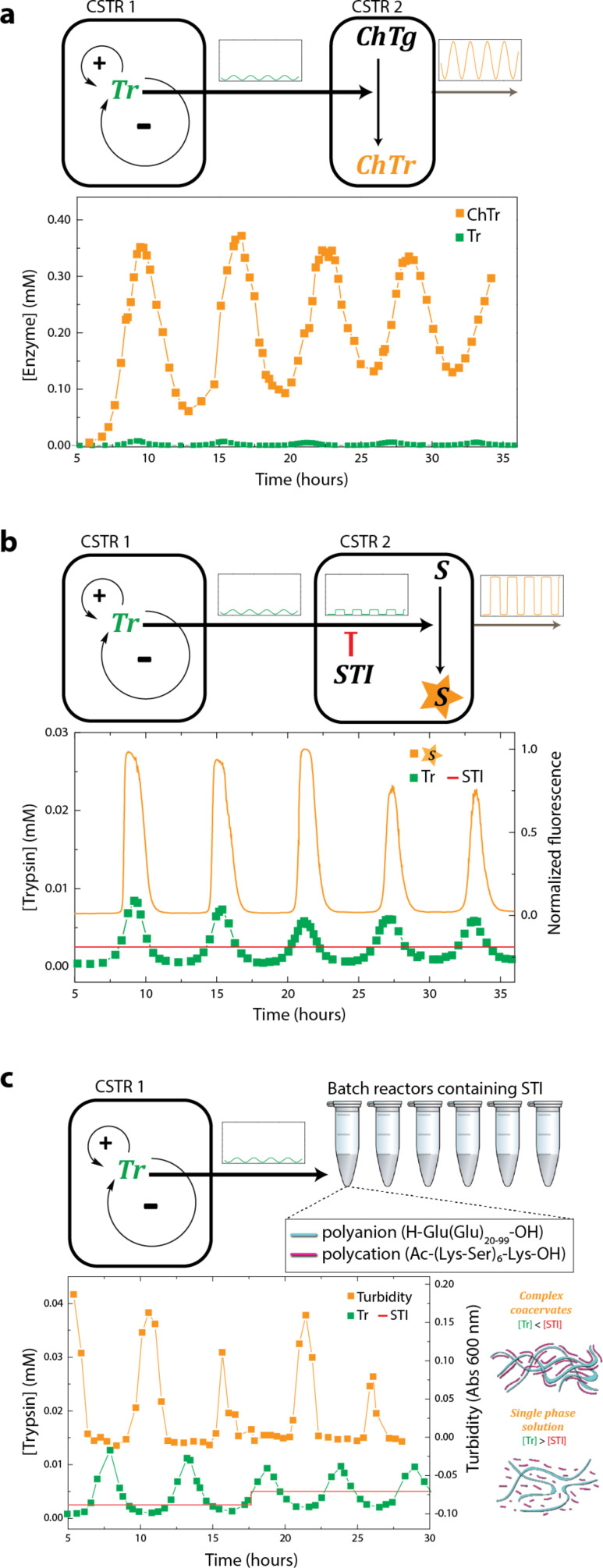
Functions obtained by linking multiple network modules in microfluidic flow reactors (depicted as CSTR 1 and 2). In each case, the oscillating catalyst concentration [Tr] from CSTR 1 is coupled to another CSTR that contains reagents producing (a) an amplification, (b) analog-to-digital conversion, or (c) a periodic control over equilibrium systems. The respective processes are: trypsin-catalyzed conversion of chymotrypsinogen (ChTg) to chymotrypsin (ChTr), trypsin-catalyzed conversion of a rhodamine substrate (S) to a fluorescent product (P) in the presence of a strong inhibitor (soybean trypsin inhibitor, STI), and trypsin-catalyzed fragmentation of a polycation (in purple) in the presence of a polyanion (in cyan). Adapted with permission from [94], copyright 2015 Nature Publishing Group.

We further processed the oscillating enzyme activity by coupling the multiple reactor modules, each with a specific chemical reaction. Figure 6 shows the feed forward designs that use an enzyme or a substrate with a high affinity to Tr in a subsequent CSTR. Figure 6a demonstrates that the initial oscillating signal can be used to create an identical timing in a subsequent enzymatic reaction. Depending on the feed concentration of chymotrypsinogen (ChTg), the initial oscillations are amplified. Similarly, the design is used to create an analog-to-digital output by introducing a trypsin inhibitor (soybean trypsin inhbitor (STI)) in the second CSTR (Figure 6b). The STI effectively thresholds the local minima in the initial oscillations, converting the initial signal into a switch-like output, creating a binary signal. Finally, we used oppositely charged polyelectrolytes to form complex coacervates in Figure 6c. Coacervates are formed in the second CSTR only in the absence of Tr, as Tr catalyzes the lysine-functionalized polycation. This demonstrates that the relatively long oscillation periods enable the construction of more complex systems capable of dynamical self-assembly. In this case, it is a dynamic self-assembly that is exactly out-of-phase with the initial oscillations.

#### Correlating the molecular structure to network behavior

**Figure 7:**
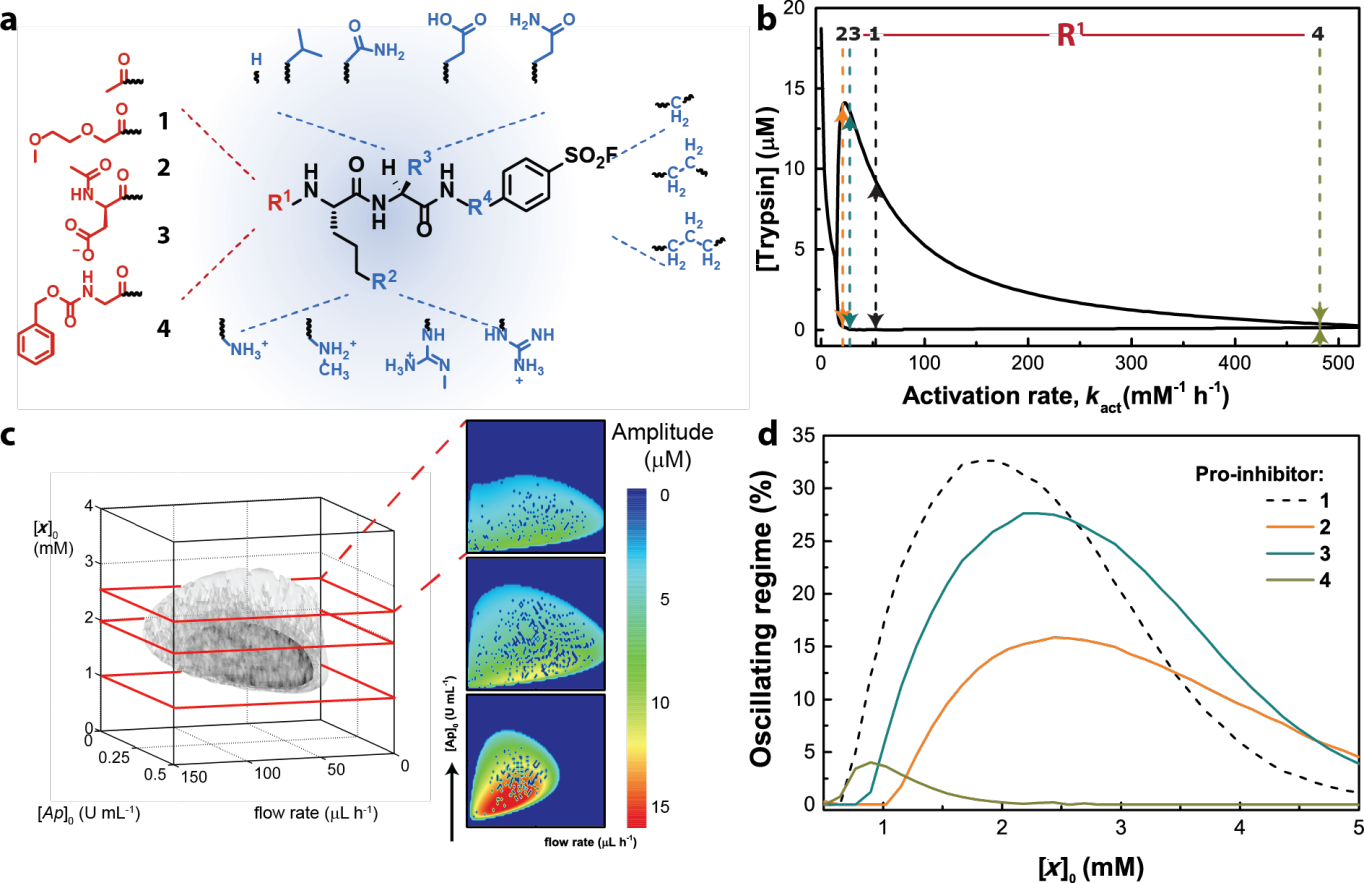
Influence of molecular structure on the properties of CRNs. (a) Molecular “Swiss army knives” showing the opportunities for tunability in different enzymatic reaction networks. (b) Bifurcation diagram with the control parameter activation rate of pro-inhibitors that were modified at position R^1^ (in red). (c) Phase plot analysis showing regimes of stable steady states are stable limit cycles, with the latter depicted as a grey volume. (d) This was used to predict the performance of the pro-inhibitor under flow conditions. Adapted with permission from [96], copyright 2015 American Chemical Society.

This design strategy enables the chemist to exploit the full power of chemical synthesis. Figure 7a depicts the synthetic sites at which the pro-inhibitor can be altered (R^1^−R^4^). In general, this allows us to create a “Swiss army knife” out of the source molecule that controls the negative feedback [30]. The possibility to make small synthetic variations provides the controllability to influence the precise rates in feedback loops. Essentially, this flexibly helped us enormously at the stage of (1) retrosynthetic screening, as well as (2) in studies correlating the molecular interactions to the behavior in networks at both regulatory as well as at the systems level.

As the networks are “synthesized”, it is in principle possible to fully know all the components and reactions in the network. We unambiguously determined the state of the system by measuring the variation in the intermediate inhibitor and the inhibitor in addition to Tr [94]. Such a molecular level understanding of networks ultimately allows us to ask questions about the relationship between individual molecules or reactions and the robustness or resilience of the network that cannot otherwise be asked in other systems [97–98].

#### Mathematical modelling

Our network is inherently nonlinear, and like most artificial complex systems, analytically unsolvable. The construction of the network combined the design of small molecules with a mathematical simulation of the complete network. Nonlinear mathematical problems that comprise more than three variables are typically difficult, if not impossible, to solve without the reduction of variables [99]. To avoid loss in chemical information, we implemented the full set of rate equations in MATLAB and COPASI that could simulate the trajectory of the individual species by the stepwise numerical integration in time. Importantly, all rate constants were determined from kinetic studies in isolated individual reactions, allowing accurate simulations to test specific details of the experiments.

We used the model to vary the rate constant that is induced by the changes to the molecular structure. First we show in Figure 7b that the tuning of R^1^ alters the steady state behavior of the CRN under identical conditions. The qualitative changes in the final state shown here are called bifurcations and show that the subtle changes in the small molecules influence the out-of-equilibrium behavior of the CRN. This analysis is expanded in Figure 7c to find the range of intrinsic (initial concentration of Ap and Pro-I), as well as a global parameters (flow rates), that we can start the experiments with. The grey volume shows the parameter space in which sustained oscillations can be found (i.e., the oscillatory regime). Typically, the CRN is robust to variations in the screened parameters but that there are differences in the size of the oscillatory regime when, for example, the feed concentration of the Pro-I ([*x*]_0_) is changed. Repeating this analysis for the different substituents depicted in Figure 7a reveals that both the size of the oscillatory regime as well as the optimal [*x*]_0_ for **1–4** differs significantly (Figure 7d) [96]. Hence, the use of mathematical modelling is an imperative tool that allows guidance to the appropriate conditions to produce sustained oscillations with **1–4**.

## Conclusion

Natural phenomena are enormously complex networks. Nonetheless, such systems remain an ensemble of smaller networks of molecules. Historically, our (dis)ability to comprehend the apparent complexity pushes science to develop theories to solve problems which were thought to be analytically unsolvable (e.g., classical or quantum mechanics) [100]. The development of the field of complex systems science will most likely follow a similar pattern, where we will get a grip on systems of increasing complexity. In this development, the rapid progress of computational methods will most probably allow us to tackle ever-larger complex systems.

**Figure 8:**
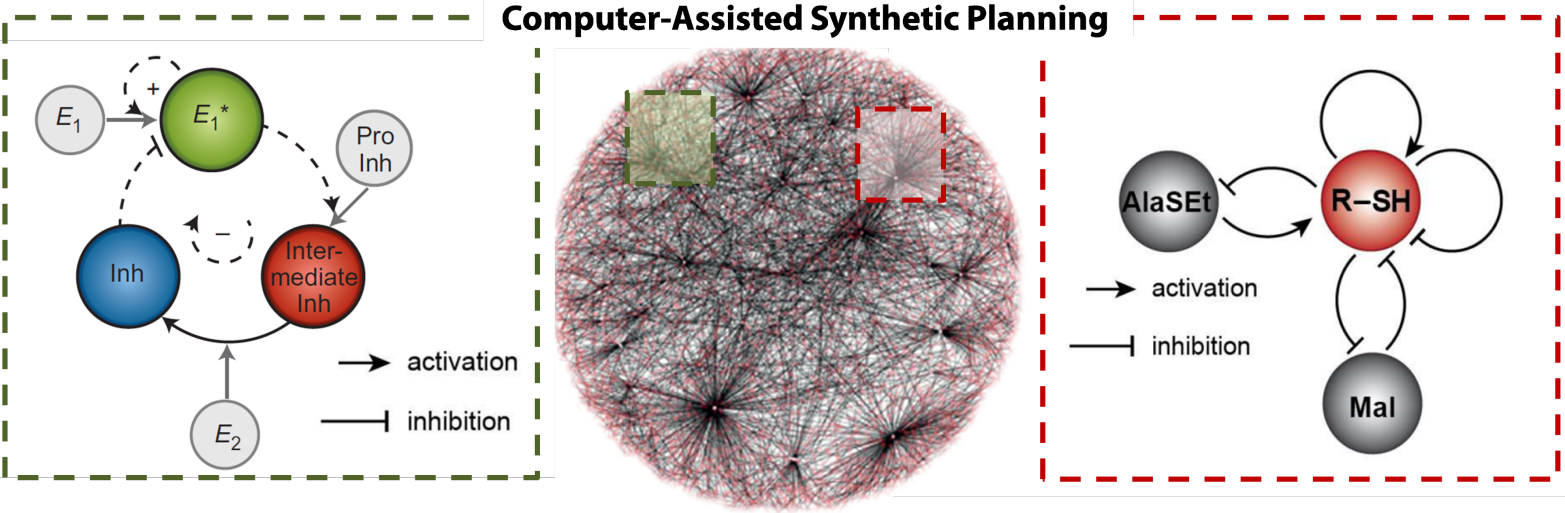
Network motifs as building blocks for the step-wise build-up of complexity. Chemical reaction networks are central to the future synthetic moves in systems chemistry. Adapted with permission from [94] copyright 2015 Nature Publishing Group, [101] copyright 2016 Nature Publishing Group, and [102] copyright 2016 Wiley-VCH Verlag GmbH & Co.

This perspective, however, urges an approach using a synthetic strategy based on the stepwise build-up of complex molecular systems. We envision the development of a toolbox that allows us to go beyond describing and understanding systems, extending to the rational design of function arising from a collection of molecular network motifs. In this respect, we believe that the complexity of future complex and functional molecular systems is by no means restricted to the network motifs and the organic chemistry we have introduced here. We conveniently made use of the specificity as well as the high turnover numbers in enzymatic reactions as a starting point to test the implementation of our design strategy. A more recent example of a chemical network capable of auto-amplification using thiols and thioesters (Figure 8) provides the ultimate proof that complex molecular systems can be designed “from scratch” [101].

## Future “synthetic moves”

We hope that the method developed here allows researchers, and especially chemists, to address important features of self-organization in complex systems. We briefly showed how the tuning of molecular structures allows one to explore the robustness of CRNs. From this perspective, other intriguing questions that still remain to be answered on the transition from non-living to living systems are: which molecules should we select from the vast pool of molecules available? Which structures allow networks to gain greater robustness and resilience? How do these systems find their steady state behavior? What trajectories do these systems take when they transition from one state to another? We fully expect these questions could move our focus from “how to build a complex system?” to “how can they emerge in a competitive or a fluctuating environment?” to “how could we employ control over a network in the presence of other networks?”.

The interactions among individual components in CRNs can change over time and space [103–107], enabling regulatory functions to emerge that are dynamic and have limited predictability. The major challenge for systems chemistry is to translate the design principles of living systems into a practical “programming language”. Computer-assisted approaches will undoubtedly aid the future plan for “synthetic moves” for complex systems [102]. Altogether, the syntheses in the context of complexity could provide a truly molecular-level insight into how chemical reactions create functionality, and ultimately, how molecules create life.
